# A thin line between conflict and reaction time effects on EEG and fMRI brain signals

**DOI:** 10.1162/imag_a_00161

**Published:** 2024-05-08

**Authors:** Ewa Beldzik, Markus Ullsperger

**Affiliations:** Institute of Applied Psychology, Faculty of Management and Social Communication, Jagiellonian University, Krakow, Poland; Institute for Medical Engineering and Sciences, Department of Electrical Engineering and Computer Science, Research Laboratory of Electronics, Massachusetts Institute of Technology, Cambridge, MA, United States; Institute of Psychology, Faculty of Natural Sciences, Otto von Guericke University Magdeburg, Magdeburg, Germany; Center for Behavioral Brain Sciences, Magdeburg, Germany

**Keywords:** conflict, response times, dorsomedial prefrontal cortex, theta, proactive control, reactive control

## Abstract

The last two decades of electrophysiological and neuroimaging research converged that the activity in the medial frontal cortex plays a pivotal role in cognitive control processes. Notably, the midfrontal theta (MFT) oscillatory EEG power as well as activity in the anterior midcingulate cortex (aMCC) or pre-supplementary motor area (preSMA) were consistently proclaimed as markers of conflict processing. However, these brain signals are strongly correlated with response time (RT) variability in various non-conflict tasks, which overshadows the true nature of their involvement. Our previous study (Beldzik et al., 2022) successfully identified these brain signals during a simultaneous EEG-fMRI experiment implementing Stroop and Simon tasks. Based on the assumption that overcoming the habitual prepotent response during high interference trials requires additional neural resources beyond simple decision variable represented in RTs, here we aim to verify if these markers exhibit a congruency effect beyond RT variations. Furthermore, we explored if these brain signals represent either proactive or reactive cognitive control mechanisms by investigating two widely known behavioral phenomena observed in conflict tasks: proportion congruency and congruency sequence effects. The results revealed partially null findings for MFT activity, yet a distinct cognitive control specialization between aMCC and preSMA. Our study provides novel evidence that the former is involved in proactive control mechanisms, possibly contingency learning, whereas the latter reflects reactive control mechanisms by exhibiting a strong congruency effect regardless of RT variation and responding to adaptive behavior.

## 1 Introduction

In our daily lives, we continuously rely on cognitive processes that allow us to adapt to changing environments and coordinate actions to optimize task goals. One of these vital processes is the human ability to detect and resolve conflict, which enables selecting relevant information despite a habitual tendency to other goal-irrelevant information. The last two decades of research in cognitive neuroscience converged that the activity in the medial frontal cortex plays a pivotal role in these functions (for reviews, seeCespón et al., 2020;Heidlmayr et al., 2020). Many EEG and fMRI studies indicated that activity in this brain region increases for high-interference trials compared to low-interference trials. However, such comparison is confounded by reaction time (RTs) differences between these trial types. Considering that the medial frontal cortex shows a strong positive correlation with RT (Cohen & Cavanagh, 2011;Domagalik et al., 2014;Yarkoni et al., 2009), it is essential to investigate the conflict-related brain activity while accounting for the RT variance.

In a simplified term, a response conflict is a competition between mutually exclusive response options. In cognitive neuroscience, such competition can be evoked by commonly used conflict tasks, that is, Stroop (Stroop, 1935), Flanker (Eriksen & Eriksen, 1974), or Simon (Simon, 1969). In these tasks, stimuli are presented with either a congruent feature (congruent trial) or a mismatched feature predisposing to a competitive response (incongruent trials). The occurrence of the conflict is manifested by the prolonged reaction times (RTs) and higher error likelihood. Several brain markers were identified that resemble this characteristic as showing greater amplitude for incongruent trials in comparison to congruent ones. These include, but are not limited to, the N2 and N450 EEG potentials (Beldzik et al., 2015a;Folstein & Van Petten, 2008), midfrontal theta (MFT) oscillatory EEG power (Asanowicz et al., 2021,^2022^;Cohen & Donner, 2013;Hanslmayr et al., 2008;Nigbur et al., 2011), as well as activity in the anterior midcingulate cortex (aMCC) or pre-supplementary motor area (preSMA) as indicated by fMRI studies (Botvinick et al., 2004;Iannaccone et al., 2015;Nachev et al., 2008;Ullsperger et al., 2014;Ullsperger & Von Cramon, 2001). These*markers of conflict processing*were linked to various subfunctions in conflict monitoring, resolution, and subsequent adaptive behavior.

However, the increased activity for more difficult trials can be explained by sustained neural activity due to the ongoing neural computations and continuous inflow of metabolic supplies. For instance, neural response in the primary visual cortex to prolonged checkerboard flashing will result in sustained visual gamma activity and linearly increasing amplitude of the BOLD fMRI signal (Engell et al., 2012;Lewis et al., 2018;Logothetis et al., 2001). The same holds for responses of varying latency to stimuli of constant duration. The amplitude of the BOLD signal in many cortical regions shows a positive correlation to RTs across various cognitive tasks (Mumford et al., 2023;Yarkoni et al., 2009). This widespread positive RT-BOLD relationship was found even in the case of fast and homogenous saccadic responses (Domagalik et al., 2014). Finally, numerous M/EEG indicators present similar tendencies for changing linearly with decision time, for example, centroparietal positivity (O’Connell et al., 2012;Twomey et al., 2015), non-phase locked MFT (Cohen & Donner, 2013;Duprez et al., 2020;Feuerriegel et al., 2021), or motor beta and gamma frequency bands (Donner et al., 2009;Fischer et al., 2018;Rogge et al., 2022).

Inspired by these proceedings,Grinband et al. (2011a)verified whether conflict-related brain activations are driven by this RT-BOLD correlation. The authors selected trials based on their RT scores and compared the two trial types when RTs were equalized or showed the opposite values of mean RTs. As a result, the aMCC activity equalized or was reversed, respectively, suggesting that this brain region is sensitive to the RT effect instead of being involved in conflict*per se*. Although this approach was questioned by others (Yeung et al., 2011), similar conclusions were drawn by another fMRI study comparing the Multi-Source Interference task with a simple RT task (Carp et al., 2012;Weissman & Carp, 2013). The authors found that the RT-dependent increase of aMCC activity in the simple RT task fully accounts for the conflict-related aMCC increase in the interference task. Our research group investigated these effects for conflict-related activity evoked by a saccadic task (Beldzik et al., 2015b). Although we carefully followed the methods used in the study byGrinband et al. (2011a), in contrast to their findings, all conflict-related brain activations, including preSMA, showed consistently greater activation for high interference stimuli. We concluded that preSMA reflects the pure congruency effect regardless of RT variations.

In a similar vein, a controversy was raised over conflict-related MFT activity. Particularly,Scherbaum and Dshemuchadse (2013)simulated theta wavelets of different lengths, corresponding to longer RTs, to quantify the influence of RT on theta power. Although the amplitude of the theta signal was kept equal for all trials, incompatible trials showed greater energy than compatible ones. In response to these arguments,Cohen and Nigbur (2013)updated the model used byScherbaum and Dshemuchadse (2013)in simulation and re-analyzed the criticized data from the Simon task, selecting trials with RTs from the same 50 ms time range. As a result, the conflict effect on MFT amplitude remained. Notably, previous studies using dipole fitting (Nigbur et al., 2011) or beamforming (Cohen & Ridderinkhof, 2013) tools have reported that the source of conflict-related MFT was estimated in the preSMA.

Thus, despite the substantial variability in methods employed, the abovementioned literature points toward a coherent picture: the aMCC activity is susceptible to the RT variability, whereas the preSMA and MFT are not. To directly (1) verify the source of MFT activity and (2) test the coherency of that picture by evoking all conflict markers in a single experiment, we conducted a simultaneous EEG-fMRI study during conflict tasks while accounting for several conditions that varied in previous reports. The results addressing the first aim were recently described byBeldzik et al. (2022). Against the original hypothesis, yet in line with previous studies exploring the MFT-BOLD amplitude correlations, we only found a negative relationship between conflict-related MFT activity and activity in midline area 9, a brain region showing conflict-sensitive deactivation.

With the coherent picture disrupted, this study aimed to verify all conflict markers regarding the simple RT-brain signals correlations (which will be called an*RT effect*from now on) and two widely known behavioral phenomena observed in conflict tasks:*proportion congruency*(Logan & Zbrodoff, 1979) and*congruency sequence*(Gratton et al., 1992;Ullsperger et al., 2005)*effects*. The proportion congruency effect is based on manipulating the frequency of congruent and incongruent trial occurrence to bias attention toward one of the stimulus features. As a result, RTs decrease for a more frequent type of trial and vice versa. Additionally, a list-wide proportion congruency effect is known to involve proactive global strategies operating before stimulus onset (Braver et al., 2007;Bugg et al., 2008,^2011^). These strategies largely reflect expectations of the upcoming stimulus type. The congruency sequence effect, on the other hand, decreases interference on a trial if a high-interference trial precedes it. Such a situation triggers reactive control, that is, short-term enhancement of the attentional set in reaction to conflict, which constitutes a fundamental mechanism for conflict adaptation (van den Wildenberg et al., 2012;Yang & Pourtois, 2022).

In our previous analysis with this dataset (Beldzik et al., 2022), we successfully identified MFT EEG activity and six independent fMRI brain networks sensitive to conflict. Here, we focused on three of those neural measures, the MFT amplitude, the aMCC, and preSMA networks’ activities, as they purport the brain markers of conflict processing commonly reported in the literature. Our goal was to verify if those markers exhibit (1) a congruency effect beyond RT variations and (2) proportion congruency and congruency sequence effects. The first goal was addressed in two fashions. First, the amplitude of each marker was compared after RT-based trial selection. We assumed that the*true*marker of conflict processing should have increased activity for high-interference trials despite similar RTs. Second, a linear mixed effect (LME) model was used to account for congruency and RT modulation in a timewise fashion. A conflict marker was expected to show significant positive LME coefficients for congruency even though the RT condition was implemented in the model. To address the study’s second goal, we ran LME separately for proportion congruency and congruency sequence effects while controlling for RT variance in the model. We assumed that a conflict marker would exhibit either a proactive or reactive cognitive control mechanism besides the primary congruency effect. Considering our previous study with oculomotor responses (Beldzik et al., 2015b), we hypothesized that preSMA is a marker that would validate these assumptions.

## 2 Methods

### 2.1 Participants

Thirty‐seven participants (mean age, 22.1 ± 2.7 years; 22 females/15 male) met the following experiment requirements: no contraindication for MRI scanning, right‐handedness (verified with the Edinburgh Handedness Inventory;Oldfield, 1971), normal or corrected‐to‐normal vision, no color-blindness (confirmed with Ishihara color vision test), no reported physical or psychiatric disorders, drug-free. Participants were informed about the procedure and goals of the study, and they gave written consent. The Bioethics Commission approved the study at Jagiellonian University. Data from two subjects were excluded during analysis due to the lack of a frontocentral component in the EEG data (see Methodssection 2.5).

### 2.2 Experimental task

**Fig. 1.**
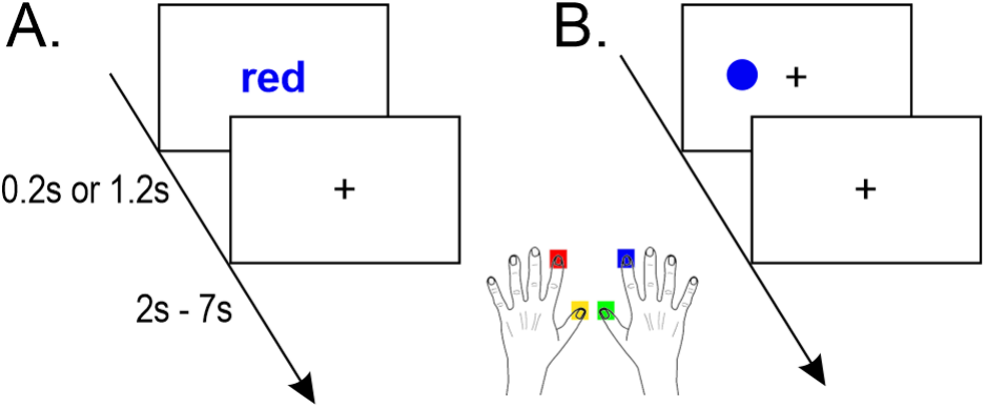
Scheme of the (A) Stroop and (B) Simon tasks.

The task was prepared and generated using E‐Prime 2.0 (Psychology Software Tools). It was presented on a 32‐inch screen placed behind the MR scanner and approximately 100 cm from the head coil. Participants performed two types of conflict-inducing tasks, that is, the Stroop (Fig. 1A) and the Simon (Fig. 1B) tasks. In the former, a stimulus was one of four color names (red, yellow, blue, or green) in Polish (Arial font, height 2°) printed in one of these colors. In the latter, a stimulus was a dot (diameter 2°) presented laterally (~22°) to the fixation sign printed in one of these four colors. Although the tasks differed in stimulus features, they had the exact instructions given to participants: “Indicate an ink color of a stimulus ignoring its other features.” Indicating a color was obtained by pressing a specified button of response grip (Nordic Neuro Lab, Bergen, Norway) using a specified finger (left index finger, left thumb, right index finger, right thumb, respectively;Fig. 1).

Response conflict was present in trials where the target response was a semantic mismatch or contralateral to the stimulus location (“incongruent trials”). Conversely, no response conflict was present in trials where the target response was a semantic match or ipsilateral to the stimulus location (“congruent trials”). The stimulus was presented for either 200 ms (“short”) or 1200 ms (“long”). In both cases, the response window was 1.2 s. A “speed up” icon was shown in case of a missing response. A fixation point (a plus sign, size 2°× 2°) was present throughout the whole experiment except for the stimuli presentation period in the Stroop task (seeFig. 1). The inter-trial time interval was randomly drawn from a uniform distribution of values in the following categories: 0.8, 1.3, 1.8, 2.3, 2.8, 5.3, and 5.8 s, resulting in an average of 3.5 s.

Each task was presented in two blocks of 50% and 20% congruency rates. Each session consisted of 60 incongruent and 60 or 240 congruent trials, respectively. These four sessions were counterbalanced between participants, with one restriction being the task type in the interleaved fashion (e.g., Stroop20%, Simon50%, Stroop50%, Simon20%). Between the sessions, subjects had an unlimited break from the task. Before beginning a new session, they were informed of the next stimulus type (word or dot) and a reminder with the response options. In total, the experiment lasted approximately 50 min and introduced 840 trials. Together, our design controlled for conflict (congruent vs. incongruent), task (Stroop vs. Simon), congruency rate (50% vs. 20%), and stimulus duration (short vs. long). The rationale for including these four conditions was to account for various task parameters, which differ in EEG and fMRI protocols.

Before the main experiment, participants undertook a training session, which included 12 centrally presented dots (neutral trials), 12 for the Simon task, and 12 for the Stroop task. If accuracy in each 12-trial run was below 90%, a subject had to redo the run.

### 2.3 EEG data acquisition and preprocessing

EEG data were recorded using an MR‐compatible EEG cap (EasyCap, Herrsching, Germany) with 63 scalp electrodes, following the extended International 10–20 system and an additional channel for recording an electrocardiogram (ECG). The ECG electrode was placed on the participants’ back under the left shoulder blade to avoid signal contamination with chest movements. The reference electrode was positioned at FCz. EEG data were recorded using Vision Recorder (Version 1.20) with a sampling rate of 5 kHz. The electrode impedances were kept below 20 kΩ. The SyncBox (Brain Products GmbH, Gilching, Germany) was used to ensure that the EEG clock was synchronized with the MR scanner clock (Mullinger et al., 2008).

The first two steps of EEG data preprocessing were conducted using BrainVision Analyzer 2.0 software (Brain Products GmbH). MR artifacts were minimized using an average artifact subtraction (AAS) technique (Allen et al., 2000). Specifically, the gradient artifact was defined as a continuous interval of 1800 ms in length, beginning at the “start volume scan” marker. An artifact template was created using a sliding average of 21 artefactual intervals. Next, datasets were downsampled to 500 Hz. The AAS technique was also applied to correct ballistocardiogram artifacts. Again, 21 pulses in the semiautomatic mode were used to create a template. Peak detection was run on the ECG channel. EEG data were then exported to EEGLAB (version 2019.1;Delorme & Makeig, 2004), excluding the ECG channel. After removing resting periods, data were filtered in a 0.5–35 Hz range (*eegfiltnew*) and re-referenced by common average.

### 2.4 EEG component selection

The EEG data were temporarily epoched to mark “bad” epochs. The criteria for a “bad” epoch included either greater than three absolute normalized channel-mean variances in the period 500 ms before to 1200 ms after the stimulus onset (Nolan et al., 2010) or greater than 150 μV absolute amplitude in ± 100 ms relative to the response onset, accounting for possible movement-related artifacts (Beldzik et al., 2019). These criteria marked 8.0% (SD 8.1%) of the trials.

The Independent Component Analysis (ICA) denoising approach was used to obtain a detectable frontocentral component for each participant (Beldzik et al., 2019;Scheeringa et al., 2011,^2016^). First, continuous EEG data were bandpass filtered in the 4–8 Hz frequency range. Following epoch extraction in 0-1200 ms post-stimulus range and exclusion of “bad” epochs and those with incorrect responses, the ICA was performed using the default, extended infomax algorithm (Lee et al., 1999). The unmixing weights were applied to the preprocessed data (i.e., before the denoising), and components were back-projected to the channel level. Applying the weights back on the original data enables time‐frequency decomposition on the full power spectrum and extended epochs instead of theta-filtered short ones. The topographical maps were visually inspected for the most prominent frontocentral component. Out of 37 participants, two did not show any frontocentral components; thus, these subjects were removed from further analyses. Epochs were extracted from −1 s to 1.8 s relative to the stimulus presentation from the selected components’ time courses.

### 2.5 EEG time-frequency analysis

The time-frequency decomposition was carried out using the complex Morlet wavelet convolution. The frequency vector comprised 60 points in the 2–30 Hz range, increasing logarithmically. Similarly, the cycle values corresponding to each frequency ranged from 2–7, increasing logarithmically. The calculated spectral power was baseline-corrected by subtracting the mean power -500 to -200 ms before stimulus onset from each time point (Duprez et al., 2020). Next, the time-frequency plots were epoched from -800 to 400 ms aligned to the response onset in 10 ms resolution. The theta power was calculated as the mean amplitude power in the 4–8 Hz range for each time point, trial, and subject and underwent further analysis discussed in detail below. All analyses conducted here included correct trials only.

### 2.6 fMRI data acquisition

MRI was performed using a 3T scanner (Magnetom Skyra, Siemens) with a 64‐channel head/neck coil. The isocenter was set 4 cm superior to the nasion to reduce gradient artifacts in the EEG data (Mullinger et al., 2011). High‐resolution, whole‐brain anatomical images were acquired using a T1‐MPRAGE sequence. A total of 176 sagittal slices were obtained (voxel size 1 × 1 × 1.1 mm^3^; TR = 2,300 ms, TE = 2.98 ms, flip angle = 9°) for coregistration with the fMRI data. Next, a B0 inhomogeneity gradient field map (magnitude and phase images) was acquired with a dual‐echo gradient‐echo sequence, matched spatially with fMRI scans (TE1 = 4.92 ms, TE2 = 7.38 ms, TR = 508 ms).

Functional T2*‐weighted images were acquired using a whole‐brain echo-planar (EPI) pulse sequence with the following parameters: 3.5 mm isotropic voxel, TR = 1800 ms, TE = 27 ms, flip angle = 75°, FOV 224 × 224 mm^2^, GRAPPA acceleration factor 2, and phase encoding A/P. Whole‐brain images (cerebellum excluded) were covered with 34 axial slices taken in an interleaved order. Due to magnetic saturation effects, each session’s first three volumes (dummy scans) were instantly discarded, resulting in two sessions of 240 and two sessions of 590 volumes for each participant.

### 2.7 fMRI data preprocessing

Preprocessing of fMRI data was conducted using Analysis of Functional NeuroImage (AFNI, version 17.3.03;Cox, 1996) and the FMRIB Software Library (FSL, version 5.0.9;Jenkinson et al., 2012). Anatomical images were skull‐stripped and coregistered to MNI (Montreal Neurological Institute) space using nonlinear transformation (*@SSwarper*). They were segmented (*FAST*) to create individual cerebrospinal fluid (CSF) masks. The first step of functional data preprocessing was to obtain the transformation matrix for motion correction (*3dvolreg*) to avoid its possible alteration by temporal interpolation applied further to fMRI data (Power et al., 2017). Next, de‐spiking (*3dDespike*) and slice timing correction (*3dTshift*) were conducted. Then, transformation matrices for coregistration of functional data to anatomical data (*align_epi_anat.py*) as well as B0 inhomogeneity derived from gradient fieldmaps (*Fugue*) were calculated. The spatial transformation was performed in one step (*3dNwarpApply*), combining all prepared matrices, that is, motion correction, anatomical co-registration, and distortion correction. The fMRI datasets were masked using a clip level fraction of 0.4, scaled to percent signal change, and the CSF signal was extracted using previously obtained individual masks. Finally, the functional images were coregistered to MNI space using the transformation matrix from nonlinear anatomical normalization.

To clear the fMRI signal from motion residuals, we applied “null” regression (*3dREMLfit*) with the pre-whitening option (using ARMA_(1,1)_model) to functional images. The model included 12 movement parameters (6 demeaned originals and 6 first derivatives), the CSF time course, and 4 or 9 polynomials as determined automatically using the “1 + int(D/150)” equation, where D is the session’s duration. The rationale for regressing the CSF signal is that this signal reflects purely physiological noises, respiratory and cardiac, and often contains motion‐related artifacts (Caballero-Gaudes & Reynolds, 2017;Power et al., 2014).

### 2.8 fMRI component selection

Our interim goal was to obtain a “functional parcellation” of the BOLD signal in the frontal cortex. To achieve that, we used a group ICA which performs data-driven decomposition into a specified number of sources (GIFT version 4.0b;Calhoun et al., 2001). Specifically, 4-dimensional fMRI datasets of all participants and runs combined are decomposed into sources characterized by a unique 3D-brain map and a single time course of its activity. Such sources are then back reconstructed for each participant and run. ICA is a powerful data-driven tool that allows separating independent BOLD signals without a priori task information even from spatially overlapping brain regions (Beldzik et al., 2013;Danielmeier et al., 2011;Eichele et al., 2008;Xu et al., 2014). In comparison to voxel-wise fMRI analyses, components’ time courses have increased temporal signal-to-noise ratio. As a result, due to extreme data reduction, testing for effects of interest can be achieved with fewer multiple comparisons and greater statistical power. To maximize separation of the sources within the medial frontal wall, we run ICA only for voxels within a mask comprising frontal, insular, and cingulate regions defined by the Harvard-Oxford cortical structural atlas (neurovault id: 1705).

An estimation of the number of components was performed using minimum description length (MDL) criteria (Y.-O. Li et al., 2007). ICA decomposition stability was validated using ICASSO (Himberg et al., 2004) with 50 random initializations of the Infomax algorithm. Data were back‐reconstructed using the default GICA option with z-scoring applied to both maps and time courses. The components’ maps were corrected with FDR at the α < .01 and inspected to identify and discard those primarily associated with artifacts representing signals from large vessels, ventricles, motion, and susceptibility (Griffanti et al., 2017;Kelly et al., 2010;Varoquaux et al., 2010). The time course of the brain’s components was interpolated to 100‐ms resolution. Next, epochs were extracted from 0 s to 10 s of the stimulus onset, and baseline corrected by subtracting the values at 0.

### 2.9 Trial selection analyses

The postprocessing of EEG and fMRI data comprised two parts. The first part focused on comparing the theta, aMCC, and preSMA amplitudes between congruent and incongruent trials while controlling for RT variance using the trial selection approach (Cohen & Nigbur, 2013;Grinband et al., 2011a). First, a classical comparison between all congruent and incongruent trials was obtained for all three conflict markers. Epochs with theta power and hemodynamic responses were averaged for each condition and compared using a paired two-tailed t-test. The p-values corresponding to each time-point obtained from these multiple t-tests were corrected with FDR at α < .05. Periods with FDR-corrected p-values were marked as grey shadings on the plots. Second, a similar analysis was conducted, and only here RTs for each trial type were equalized. Selection of trials was performed by normalizing RTs for each participant and including congruent trials within -0.6–1.3 z-values and incongruent trials within -1.5–1.4 z-values. These values were estimated to ensure a nonsignificant RT difference between the congruency conditions and a maximal number of trials for the comparison (58% of congruent and 78% of incongruent trials remained after selection). Third, data were separated into quantiles based on the normalized RT values of incongruent trials, and congruent trials were selected to match RT values in each bin. This last comparison was conducted only for theta and BOLD peak amplitudes to compensate for the lost statistical power due to trial selection.

### 2.10 LME analyses

The second part of EEG and fMRI data analyses was applied using a linear mixed-effect (LME) model to investigate neural processes related to conflict adaptation on a trial-by-trial level. A matrix consisting of all subjects combined was created that included subject ID, trial type, previous trial type, congruency ratio, and raw and normalized RTs, and was merged with the corresponding brain measure under investigation. All LME analyses were conducted using*fitlme*Matlab (2021b)function (with default parameters). The models were designed with a maximal random-effects structure (Barr et al., 2013). Particularly, theta, aMCC, and preSMA amplitudes underwent the LME model to account for (1) RT variability, (2) proportion congruency effect, and (3) congruency sequence effect. The model is ideal for capturing small effect sizes as it enables the inclusion of single-trial measurements in one group analysis. It has proven useful in previous studies (Beldzik et al., 2019,^2022^). The RT model was a simple formula applied to all three brain measures at each time point within their epochs:

brain measure ~ type + RT + (1 + type +RT​|Subject)

We assumed that if the estimates for conflict types were significant despite inclusion of RT regressor in the model, it would speak in favor of the marker of conflict. Otherwise, this brain marker primarily represents the time of neural computations or could simply be confounded by RTs due to spurious correlations with the hemodynamic response model (Mumford et al., 2023). Obtained confidence intervals for multiple time points were adjusted to an alpha value calculated separately for each brain measure using FDR correction.

Next, we examined if these brain measures showed more sophisticated conflict-related effects, that is, proportion congruency and congruency sequence effects. To maximize their sensitivity, we marked the time point with maximal congruency effect for each brain measure and extracted the values in the 100 ms time range around it. Such values underwent LME models with the following formulas:

brain measure ~  type*rate+RT+ (1 + type*rate +  RT​|Subject)

for the proportion congruency effect, and

brain measure ~  type*p.type+RT+  (1  +type*p.type+ RT​|Subject)

for the congruency sequence effect, where*p.type*denotes the previous trial type. Brain measures, as well as RT values, were normalized within-subject (using z-score) before running each model. Additionally, RT values underwent similar LME analyses (without RT regressor) to verify the behavioral effects in the data.

Additionally, to improve the interpretation of the interaction effects obtained with the above models, we conducted time point-wise LME analyses with two conditions of interest combined (e.g., congruent-20% rate, incongruent-20% rate, etc). Specifically, the LME formulas were as follows:

brain_measure ~  −1 + con_20+con_50 +incon_20                                                 +incon_50+RT+(−1+con_20                                             +con_50+incon_20+incon_50 +RT ​| Subject)

brain measure ~ −​1+con_p.con+con_p.incon                                                            +incon_p.con + incon_p.incon + RT                                                            + (−1 + con_p.con + con_p.incon                                                 + incon_p.con + incon_p.incon + RT ​| Subject)

The intercept term was removed from the model (the “-1” annotation) to address the issue of full-rank matrix design. These were compared to the mean hemodynamic time series for conditions combined. In both cases, time series were normalized (z-scored) and baseline corrected at time point 0 for compatibility with the previous LME analyses. Notably, these analyses were performed for visualization purposes instead of rigorous statistical testing.

## 3 Results

### 3.1 Defining conflict markers of interest

**Fig. 2.**
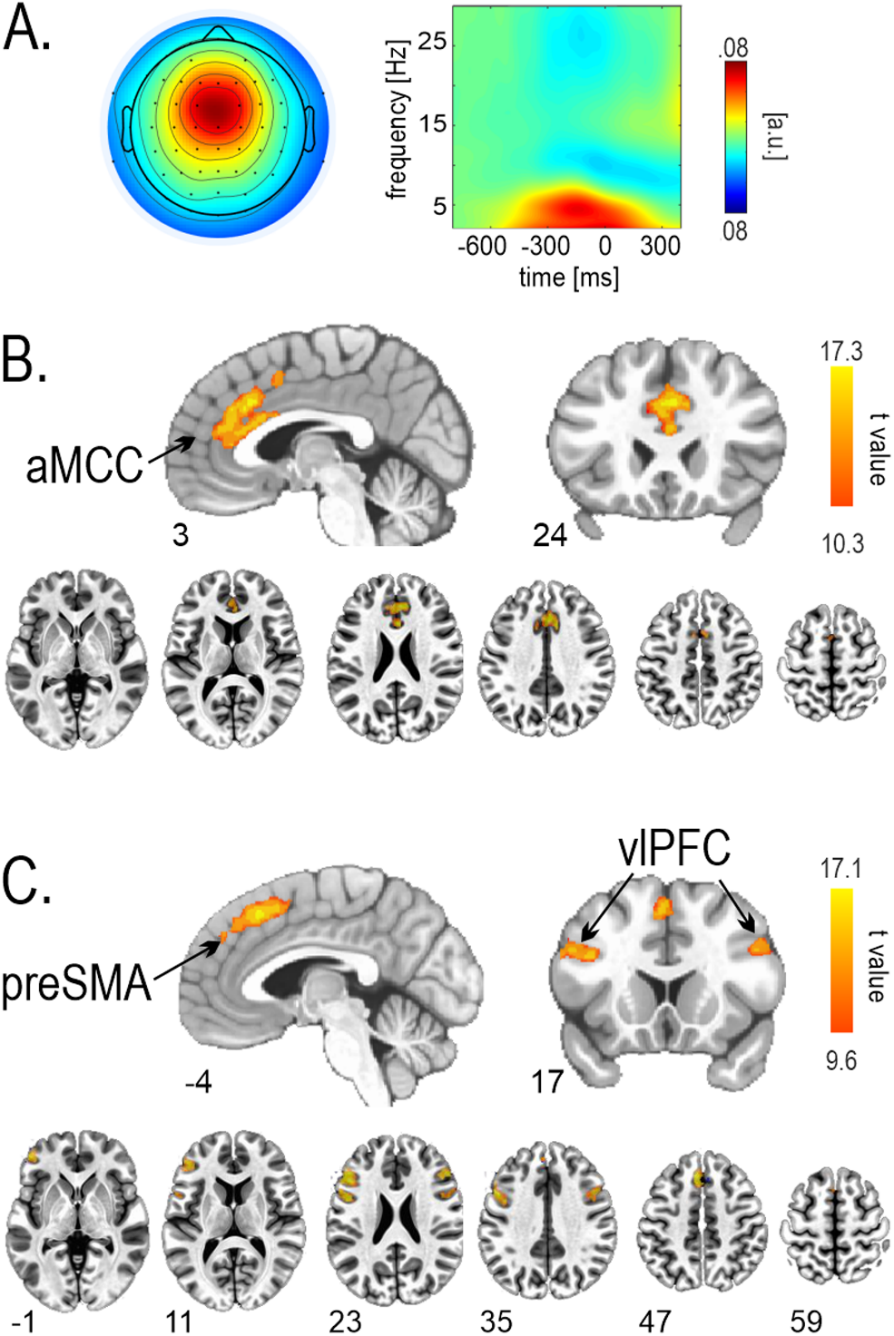
Conflict markers of interest—maps at the group level. (A) Midfrontal EEG component topography (left) and response-locked spectral power (right). fMRI components corresponding to (B) aMCC and (C) preSMA. preSMA = pre-supplementary motor area. aMCC = anterior midcingulate cortex. vlPFC = ventrolateral prefrontal cortex.

A comprehensive description of the results of the EEG and fMRI data analysis was presented in our previous work (Beldzik et al., 2022). Here, we focus only on three conflict markers identified before and widely referenced in the literature, that is, (1) response-locked theta power, (2) aMCC, and (3) preSMA hemodynamic responses. The selected EEG components with frontocentral topography showed a maximum at the FCz channel and pronounced activity in the response-locked theta power (Fig. 2A). The fMRI results revealed two distinct components involving the cortical loci of interest (Table 1;Fig. 2B). The aMCC component covered a single region, whereas the preSMA was functionally coupled to the bilateral ventrolateral prefrontal cortices. For simplicity, we shall refer to this component as the preSMA network. Notably, in the study bySallet et al. (2013), the authors investigated the resting-state connectivity of all subregions within the medial frontal wall. They found that the preSMA was coupled to the caudal ventral prefrontal cortex centered at [49, 31, 19]. These coordinates are in close proximity to the coordinates of the ventrolateral prefrontal cortex found here [49, 31, 22].

**Table 1.** Brain regions corresponding to fMRI brain components.

Label	Region	Side	x	y	z	t
aMCC	cingulate gyrus	M	3	24	33	16.7
preSMA	pre-supplementary motor area	M	-4	24	50	15.7
	inferior frontal sulcus	L	-49	-10	33	16.1
		R	49	31	22	13.3

Note. R = right; L = left; M = medial; A = area; x.y.z coordinates are provided in MNI space. preSMA = pre-supplementary motor area. aMCC = anterior midcingulate cortex.

### 3.2 Trial selection results

Participants (N = 35) committed 5.9% (SD 3.9%) erroneous responses and 3.9% (SD 3.2%) omissions. All results reported here are based on correct trials only. 7.9% (SD 7.6%) of the trials were marked as “bad epochs” during EEG preprocessing and were also removed. Thus, further analyses were conducted using 83.3% of the trials, that is, 695.0 (SD 79.8) trials on average for each participant.

**Fig. 3.**
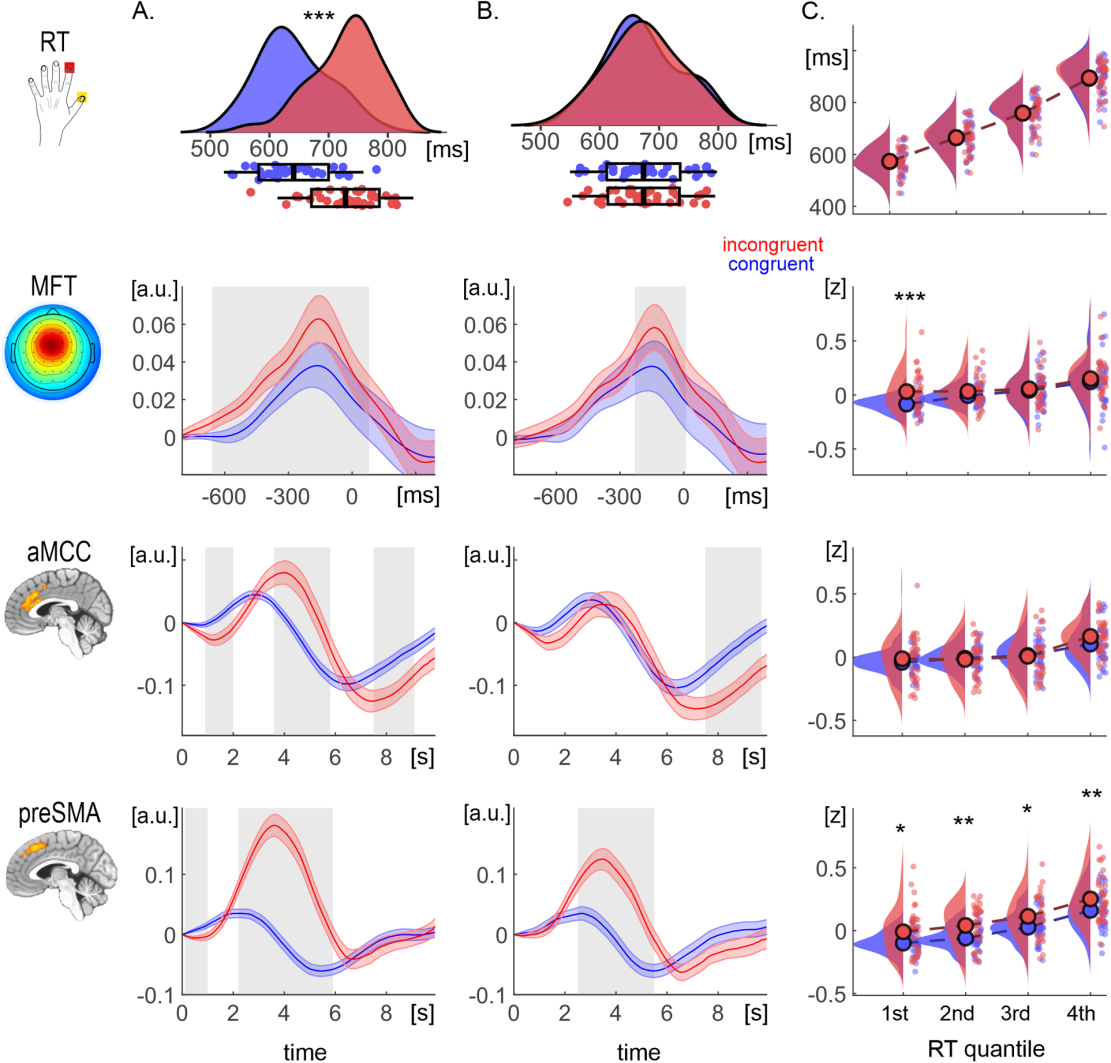
The results of trial selection analyses aimed to verify the RT effect. Congruency effect for the amplitude of the MFT, aMCC, and preSMA in the case for (A) all trials, (B) trials with equalized RT, and (C) quantiles with equalized RT (peak activity only). The top row presents the corresponding RT values. Ribbons denote standard errors. The shaded areas represent FDR-corrected significant t-tests (p_cor_< .05). ***p < .001; **p < .005; and *p < .05. MFT = midfrontal theta; aMCC = anterior Midcingulate Cortex; preSMA = pre-supplementary motor area.

In line with the assumption of conflict processing, congruent and incongruent trials differed substantially in their mean RTs (congruent: 641.0; SD 59.0 ms; incongruent 728.4, SD 57.4 ms; t(34) = 18.8, p < .001; d_cohen_= 3.2). For this comparison, all three brain measures showed significantly greater activity for incongruent trials than for congruent ones (Fig. 3A). In the next comparison, we selected trials to equalize their group-mean RTs (congruent: 673.6 ms; SD 62.1 ms; incongruent 673.8 ms; SD 60.6 ms; t = 0.1, p = .92; d_cohen_= 0.02). As a result, conflict-related differences in theta and preSMA activity weakened yet remained significant (Fig. 3B). In contrast, the difference between congruent and incongruent trials in the case of aMCC activity vanished at the peak of hemodynamic response but remained significant at the undershoot. The third comparison was conducted between quantile bins based on the normalized RTs. The results revealed that the only brain measure which showed persistent conflict-related sensitivity at each bin was the preSMA (Fig. 3C). The midfrontal theta, on the other hand, showed a profound congruency effect but only in the case of the fastest trials.

### 3.3 LME results

**Fig. 4.**
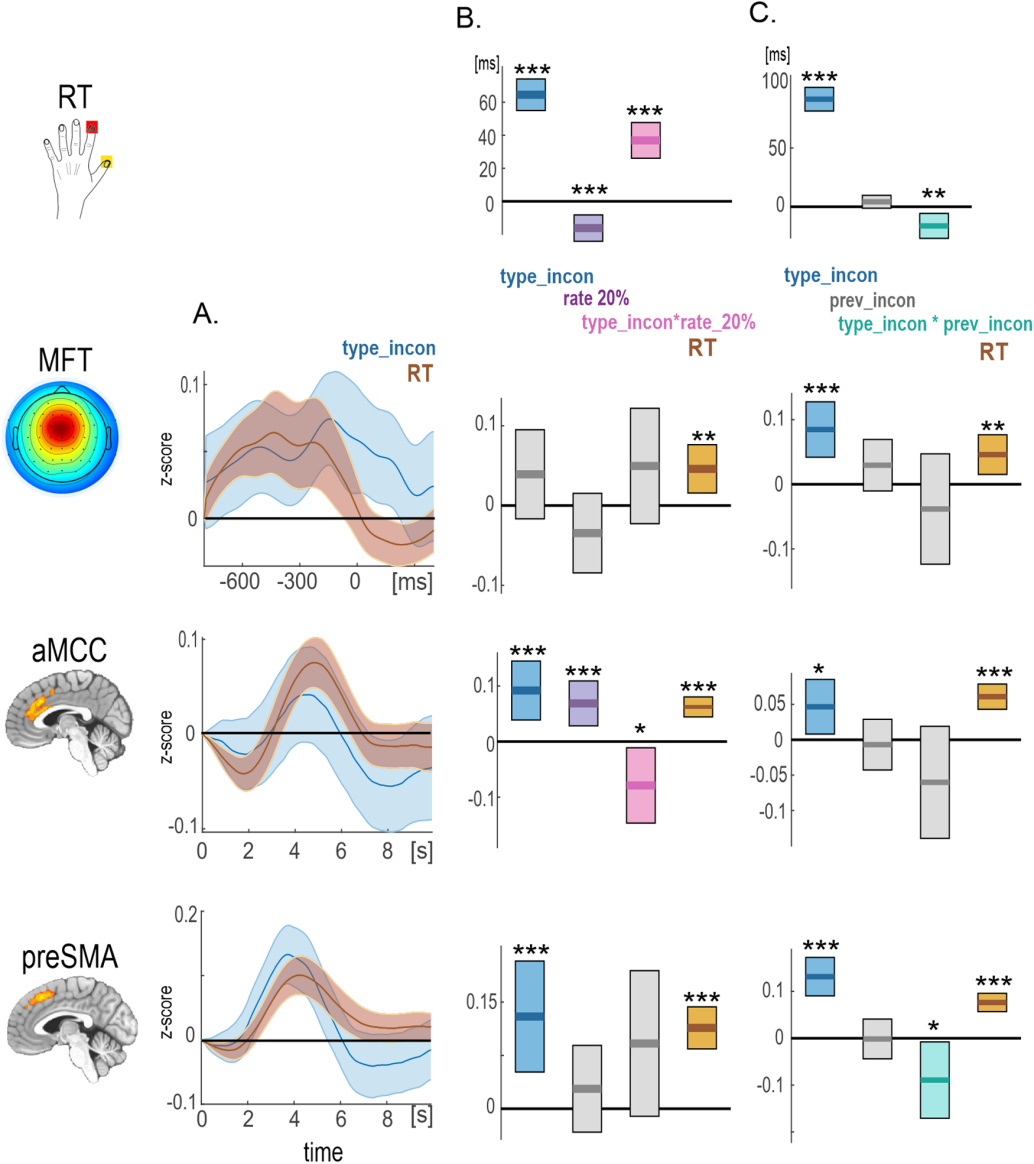
The results of LME analyses aimed to verify the RT, proportion congruency, and congruency sequence effects. (A) Estimates for the trial type and normalized reaction times (RTs) effects predicting the amplitude of the MFT, aMCC, and preSMA in a point-by-point fashion. Incon/con labels refer to the “type” condition only. (B) Estimates for the raw RTs (1st row) and the amplitude of each brain measure (2nd— 4th rows) at the peak of congruency effects exploring (B) the proportion congruency and (C) congruency sequence effects. Ribbons denote confidence intervals. ***p < .001; **p < .005; and *p < .05. MFT = midfrontal theta; aMCC = anterior Midcingulate Cortex; preSMA = pre-supplementary motor area.

In the second part of data postprocessing, we conducted data analyses with three approaches using the LME model. The first LME analysis investigating congruency and RT effects at each time point of brain amplitudes revealed a general temporal overlap of the two effects (Fig. 4A), which suggests that all brain signals met our first conflict assumption. Further LME analyses were conducted only for the amplitude values at the time point with maximal congruency effects. Such selection introduces circularity but is based on the assumption that the proportion congruency and congruency sequence effects are tightly linked to the congruency effect. The maximal congruency effects were observed at -135 ms before the response in theta activity and 4.5 s and 4.0 s after the stimulus in aMCC and preSMA, respectively.

The second LME analysis investigating the proportion congruency effect was applied to RT scores and three brain measures (Table 2;Fig. 4B). In general, responses were considerably faster in blocks with a 20% congruency rate than in 50% blocks, indicating the facilitation process related to the sequence of congruent trials. Most importantly and in line with the proportion congruency effect, responses for incongruent trials were slower (in addition to the general conflict effect) in 20% blocks than congruent trials in 50% blocks, as indicated by the interaction term. This finding can be linked with increased interference for 20% of blocks. The MFT activity showed a general decrease in 20% blocks compared to the 50% blocks. Interestingly, the aMCC activity exhibited a pronounced increase in the high-congruent block in general and a decrease for high-interference trials in that block as compared to the low-congruent block and low-interference trials, respectively.

**Table 2.** Results of LME analysis investigating the proportion congruency effect.

Dependent	Predictor	Estim	SE	t	DF	p-Value	Lower	Upper
RT (ms)	intercept	649.98	9.95	65.32	20246	.000	630.48	669.49
	type_incon	64.54	4.88	13.24	20246	.000	54.99	74.10
	rate_20%	-16.23	4.07	-3.98	20246	.000	-24.21	-8.24
	type_incon:rate_20%	36.90	5.51	6.70	20246	.000	26.10	47.70
MFT	intercept	0.01	0.02	0.33	20245	.739	-0.03	0.05
	type_incon	0.04	0.03	1.38	20245	.169	-0.02	0.09
	rate_20%	-0.03	0.03	-1.36	20245	.175	-0.08	0.02
	type_incon:rate_20%	0.05	0.04	1.34	20245	.179	-0.02	0.12
	RT	0.05	0.02	2.98	20245	.003	0.02	0.08
aMCC	intercept	-0.06	0.02	-3.46	20245	.001	-0.10	-0.03
	type_incon	0.09	0.03	3.40	20245	.001	0.04	0.15
	rate_20%	0.07	0.02	3.33	20245	.001	0.03	0.11
	type_incon:rate_20%	-0.08	0.03	-2.28	20245	.022	-0.15	-0.01
	RT	0.06	0.01	6.78	20245	.000	0.04	0.08
preSMA	intercept	-0.05	0.02	-2.46	20245	.014	-0.08	-0.01
	type_incon	0.09	0.03	3.26	20245	.001	0.03	0.14
	rate_20%	0.02	0.02	0.90	20245	.367	-0.02	0.06
	type_incon:rate_20%	0.06	0.03	1.76	20245	.079	-0.01	0.13
	RT	0.08	0.01	7.53	20245	.000	0.06	0.10

Note. Estim = parameter estimate. SE = standard error. DF = degrees of freedom. Lower/Upper = lower/upper confidence interval. MFT = midfrontal theta; aMCC = anterior midcingulate cortex; preSMA = pre-supplementary motor area.

The final LME analysis investigating the congruency sequence effect was applied to brain measures and raw RT scores (Table 3;Fig. 4C). In line with the congruency sequence effect, when preceded by an incongruent trial, responses to incongruent stimuli speeded profoundly. Also, there was a marginal post-conflict slowing of congruent trials. These results suggest behavioral adaptation and loss of interference following incongruent trials. Regarding the brain measures, only the activity in preSMA showed this interaction effect as significant (Fig. 4C, bottom row). Notably, all LME results reported here show similar results even when normalized RTs are included in the model. Full model results are provided in theSupplementary Data file.

**Table 3.** Results of LME analysis investigating the congruency sequence effect.

Dependent	Predictor	Estim	SE	t	DF	p-Value	Lower	Upper
RT (ms)	intercept	635.57	10.15	62.62	20246	.001	615.67	655.46
	type_incon	91.47	5.13	17.83	20246	.001	81.42	101.52
	prev_type_incon	4.25	2.80	1.52	20246	.129	-1.23	9.74
	type_incon:prev.type_incon	-16.26	5.41	-3.01	20246	.003	-26.86	-5.66
MFT	intercept	-0.03	0.01	-2.69	20245	.008	-0.05	-0.01
	type_incon	0.08	0.02	3.88	20245	.001	0.04	0.13
	prev_type_incon	0.03	0.02	1.45	20245	.147	-0.01	0.07
	type_incon:prev.type_incon	-0.04	0.04	-0.88	20245	.379	-0.12	0.05
	RT	0.05	0.02	2.9	20245	.003	0.02	0.08
aMCC	intercept	-0.01	0.01	-0.69	20245	.487	-0.03	0.01
	type_incon	0.05	0.02	2.37	20245	.018	0.01	0.09
	prev_type_incon	-0.01	0.02	-0.37	20245	.707	-0.04	0.03
	type_incon:prev.type_incon	-0.06	0.04	-1.49	20245	.137	-0.14	0.02
	RT	0.06	0.01	6.70	20245	.001	0.04	0.08
preSMA	intercept	-0.03	0.01	-2.75	20245	.006	-0.05	-0.01
	type_incon	0.13	0.02	6.29	20245	.001	0.09	0.17
	prev_type_incon	0.00	0.02	-0.07	20245	.943	-0.04	0.04
	type_incon:prev.type_incon	-0.09	0.04	-2.15	20245	.032	-0.17	-0.01
	RT	0.08	0.01	7.65	20245	.001	0.06	0.10

Note. Only significant estimates are presented. Estim = parameter estimate. SE = standard error. DF = degrees of freedom. Lower/Upper = lower/upper confidence interval. MFT = midfrontal theta; aMCC = anterior midcingulate cortex; preSMA = pre-supplementary motor area.

**Fig. 5.**
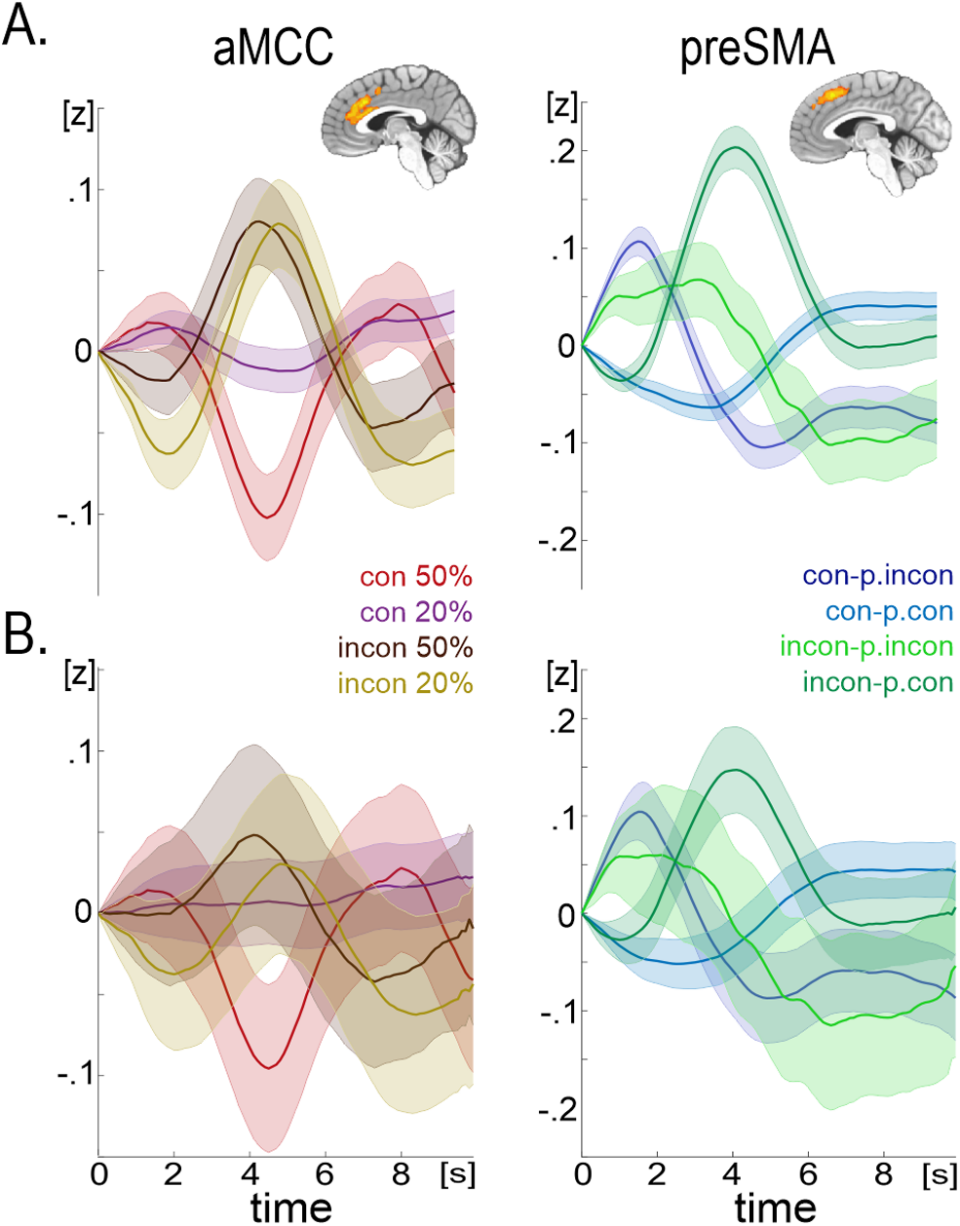
Mean hemodynamic responses (A) and estimates from the LME models (B) for the combination of conditions of interest to improve interpretation of the significant interaction effects that were detected using the LME analyses presented inFigure 4B-C. Shaded bars represent standard errors (A) or confidence intervals (B).

To improve the interpretation of the LME results with significant interaction effects, we additionally calculated (1) mean hemodynamic responses for the two conditions of interest combined (e.g., congruent-20%, incongruent-20%, etc.,Fig. 5A) and (2) LME models for these combined conditions (see Methodssection 2.10for details,Fig. 5B). The two methods are equivalent, yet the latter is improved as it accounts for the RT variance in the brain signals as well as variations across individuals.

## 4 Discussion

The goal of the study was to explore the preservation of the congruency effect, and other conflict-related effects, after controlling for RT variance in three commonly reported conflict markers. The results obtained presented a complex picture. MFT showed partial preservation to congruency effect similar to the aMCC brain activity, but the latter brain marker exhibited a profound congruency proportion effect, yet distinct from RT outcomes. In contrast, the preSMA brain network showed the clearest picture in preserving congruency effect and the only marker sensitive to the congruency sequence effect that closely aligns with behavioral effects.

The functional relevance of the MFT role in cognitive control has been debatable by previous EEG-fMRI aiming at linking MFT to brain regions actively engaged in this process yet finding only a negative correlation to the default mode network activity during the resting state (Prestel et al., 2018;Scheeringa et al., 2008), working memory (Scheeringa et al., 2009), and decision (Algermissen et al., 2021) tasks. In line with those studies, our previous analyses of the same dataset found a consistent negative correlation of conflict MFT with the BOLD amplitude in the midline area 9, a brain region showing conflict-sensitive deactivation and omission-preceding activation (Beldzik et al., 2022). We concluded that the negative relationship of this brain area with conflict theta suggests that MFT plays a role in an active inhibition of the self-referential and mind-wandering processes that may otherwise distract participants from the task at hand and lead to omission error (Durantin et al., 2015;C. S. R. Li et al., 2007).

The results obtained here regarding RT, proportion congruency, and congruency sequence effects on MFT activity are not against that conclusion. In line withCohen & Nigbur (2013), the MFT showed increased activity for incongruent trials in comparison to congruent ones for trials with equalized RTs (Fig. 3B). However, more detailed analysis with multiple RT bins indicated the difference only for the case of the fastest trials (Fig. 3C). Next, the point-by-point LME analysis revealed that congruency condition affects MFT power significantly even when controlling for RT variance (Fig. 4A). The following LME analyses showed a lack of MFT findings regarding both effects of interest (Fig. 4B, C). On the one hand, the null congruency sequence effect is in opposition to previous EEG studies (Gyurkovics & Levita. 2021;van Driel et al., 2015), and it may be a result of the generally low quality of EEG data recorded in the magnetic field environment. On the other hand, the MFT results obtained here provide little evidence for theta reflecting a conflict-related process, which is in line with several recent findings indicating that the role of MFT is not conflict-specific (Kaiser & Schütz-Bosbach, 2021;Kaiser et al., 2023). MFT may be involved in the active inhibition of self-reflective cognition that may otherwise disrupt optimal performance (Beldzik et al., 2022), reflect passive cortical disengagement, where an entire network or brain area ceases to receive inputs and essentially goes in “standby," similar to occipital alpha waves (Snipes et al., 2022) or linked to task-independent processing efficiency (Weigard & Sripada, 2021). Such inhibitory mechanisms may still be considered as control signals if disengagement of disruptive processes would lead to optimizing performance or behavioral adjustments as was reported for MFT in the literature (Kaiser et al., 2022;Ullsperger & Danielmeier, 2016;Wessel, 2018).

The aMCC brain activity showed consistent sensitivity to the RT effect. In line withGrinband et al. (2011a), the conflict effect disappeared when aMCC activity was compared to trials with equalized RTs (Fig. 3B-C) as well as congruency regressor in point-by-point LME analysis was nonsignificant when accounting for RT in the model (Fig. 4A). However, aMCC showed a prominent proportion congruency effect, yet opposite direction to RTs scores (Fig. 4B). Specifically, a congruency effect can be detected in aMCC but is far greater in 50% congruency ratio block than in 20% blocks (Fig. 5). This finding fits well with the theoretically and behaviorally outlined accounts of proactive control mechanisms in conflict, particularly contingency learning (Braem et al., 2019;Bugg, 2017;Bugg & Crump, 2012;Schmidt, 2013). The aMCC seems to play a role in anticipating when the next incongruent stimulus will appear, the process that leads to enhanced activation for congruent trials in blocks with a congruency ratio above the chance level, that is, 20% blocks in this study (Fig. 5). This finding fits well with the idea that aMCC represents the value of expectancy violation (Vassena et al., 2020), that is, when incongruent trials are expected and a congruent one comes, this is surprising and may trigger some adjustments.

Notably, none of the brain measures explored here explain the behavioral effects of proportion congruency manipulation. The reason for this may lie in the fact that a greater congruency effect on RTs in blocks with a 20% congruency ratio is linked to motor slowing on anticipated conflict trials (in contrast to randomly occurring ones), a proactive cognitive process that was previously shown to modulate mostly motor and sensory systems (Kaiser & Schütz-Bosbach, 2019). Similar to the Kaiser and Schütz-Bosbach study, we did not find MFT involvement in this phenomenon. Further research should be designed to determine if these sensory systems are driven by aMCC proactive activity.

Finally, these effects were tested on a network, involving preSMA and bilateral inferior frontal sulci. Due to the highly coherent and impossible to isolate BOLD activity in those brain regions, any inferences made to the network apply to all of them, yet we refer to the network “preSMA” for simplification. The preSMA showed a robust congruency sequence effect despite fully accounting for RT variance in the data. In line with our previous study using oculomotor responses (Beldzik et al., 2015b), preSMA increased for incongruent trials in comparison to congruent for all RT bins (Fig. 3C). Also, the congruency effect showed robust significance in the LME model (Fig. 4A). Thus, these results converge to a clear conclusion that conflict-related activity in preSMA is independent of RT variance.

This conclusion has important implications in a debate over the “concept of conflict” introduced byGrinband et al. (2011a). The fact that RT variance could explain the conflict-related activity in aMCC activity had posed a serious challenge to the conflict-monitoring hypothesis (Botvinick et al., 2004).Yeung et al. (2011)replied that slow RTs are a stronger indication of conflict than the stimulus category. We argue that if the conflict was better “tracked” by RT than stimuli categorization and, as a result, incongruent trials with relatively fast responses may have not triggered any conflict, why would the preSMA and inferior frontal sulci respond stronger to such trials? Our findings prove that conflict is a process that engages additional neural resources to overcome the biased response even when RTs were not lengthened. In line withGrinband et al. (2011b), we argue that defining conflict as “any sensorimotor or cognitive process that lengthens RT” trivializes the idea of conflict and weakens its usefulness as a psychological construct. With such a definition, it would be impossible to differentiate conflict from any other sensorimotor, memory, or attentional processes. Together, our results strongly recommend accounting for RT variance in any cognitive task when exploring brain signals underlying the cognitive processes of interest.

Finally, we found that preSMA showed decreased activity in the incongruent trial that followed another incongruent trial. The congruency sequence effect was observed even when controlling for RT variance (Fig. 4C). The congruency sequence effect is typically thought to measure a short-lived, reactive adaptation to a just-experienced conflict between competing response representations (Braem et al., 2019;van den Wildenberg et al., 2012;Yang & Pourtois, 2022). Thus, our findings indicated that preSMA is involved in conflict adaptation. Notably, in a recent study byFu et al. (2022), the spiking of neuronal assemblies was directly recorded in the medial frontal cortex of epilepsy patients performing a Stroop task. Although the authors found that both aMCC and preSMA areas independently supported compositional conflict coding, the former demonstrated pre-response conflict information first, whereas the latter exhibited post-conflict information first. Their results obtained here align well with the proactive and reactive functional distinction of aMCC and preSMA brain regions.

## 5 Conclusions

The study verified three highly replicable conflict markers for congruency, proportion congruency, and congruency sequence effects while controlling for RT variance. The pre-response MFT EEG activity showed a lack of congruency effect when the two trial types had no differentiating RTs, except for the fastest response bin. The fMRI data revealed a distinct cognitive control specialization between the two brain regions of interest. The aMCC activity was increased for blocks where congruent trials occurred five times more often than incongruent ones. Although its activity showed a general increase for high-interference trials compared to low-interference, this effect was attenuated in 20% congruency ratio blocks. In contrast, preSMA manifested a significant congruency sequence effect even though RTs were included in the model. Together, our results indicate that aMCC is involved in proactive expectation of rare stimuli, whereas preSMA is responsible for reactive control by resolving the conflict and adapting to it both on the behavioral and neural levels.

## Supplementary Material

Supplementary Material

## Data Availability

The source data are also publicly available athttps://osf.io/cx8a9/. All codes generated for this study’s analyses are publicly available athttps://github.com/ewabeldzik/conflict_RT_effects
