# Supplementary material for: A thin line between conflict and reaction time effects on EEG and fMRI brain signals
Source: Imaging Neurosci (Camb). 2024 May 8;2:imag-2-00161. doi: 10.1162/imag_a_00161 (PMC12247625; doi:10.1162/imag_a_00161)
Supplement: Supplementary Material [file imag_a_00161-supp.pdf]

Supplementary Data

Table of Contents

1. Full LME results presented in Fig 4B and Table 2 for 1
1a. RT 1
1b. MFT 2
1c. aMCC 3
1d. preSMA 4
2. Full LME results presented in Fig 4C and Table 3 for 5
2a. RT 5
2b. MFT 6
2c. aMCC 7
2d. preSMA 8

1. Full LME results presented in Fig 4B and Table 2 for

1a. RT

Linear mixed-effects model fit by ML

Model information:

Number of observations	20250
Fixed effects coefficients	4
Random effects coefficients	140
Covariance parameters	11

Formula:

stimRT ~ 1 + type*freq + (1 + type*freq | Subject)

Model fit statistics:

AIC	BIC	LogLikelihood	Deviance
2.571e+05	2.5722e+05	-1.2854e+05	2.5707e+05

Fixed effects coefficients (95% CIs):

Name	Estimate	SE	tStat	DF	pValue	Lower	Upper
{'(Intercept)' }	649.98	9.9506	65.321	20246	0	630.48	669.49
{'type_incon' }	64.544	4.8752	13.239	20246	0	54.988	74.099
{'freq_congr20' }	-16.229	4.0732	-3.9842	20246	6.7942e-05	-24.212	-8.2447
{'type_incon:freq_congr20'}	36.897	5.5103	6.6959	20246	2.1997e-11	26.096	47.697

Random effects covariance parameters (95% CIs):

Group: Subject (35 Levels)

Name1	Name2	Type	Estimate	Lower	Upper
{'(Intercept)' }	{'(Intercept)' }	{'std' }	56.907	44.249	73.187
{'type_incon' }	{'(Intercept)' }	{'corr'}	-0.29477	-0.67399	0.2074
{'freq_congr20' }	{'(Intercept)' }	{'corr'}	-0.06057	-0.49722	0.40057
{'type_incon:freq_congr20'}	{'(Intercept)' }	{'corr'}	0.1233	-0.48393	0.65038
{'type_incon' }	{'type_incon' }	{'std' }	19.328	10.995	33.977
{'freq_congr20' }	{'type_incon' }	{'corr'}	-0.41246	-0.89319	0.50822
{'type_incon:freq_congr20'}	{'type_incon' }	{'corr'}	0.12602	-0.79602	0.87193

{'freq_congr20' }	{'freq_congr20' }	{'std' }	17.239	10.968	27.095
{'type_incon:freq_congr20'}	{'freq_congr20' }	{'corr'}	0.40272	-0.68343	0.93406
{'type_incon:freq_congr20'}	{'type_incon:freq_congr20'}	{'std' }	17.474	7.5854	40.253

Group: Error

Name	Estimate	Lower	Upper
{'Res Std'}	137.3	135.96	138.64

1b. MFT

Linear mixed-effects model fit by ML

Model information:
Number of observations 20250
Fixed effects coefficients 5
Random effects coefficients 175
Covariance parameters 16

Formula:
MFT ~ 1 + normRT + type*freq + (1 + normRT + type*freq | Subject)

Model fit statistics:

AIC	BIC	LogLikelihood	Deviance
57302	57468	-28630	57260

Fixed effects coefficients (95% CIs):

Name	Estimate	SE	tStat	DF	pValue	Lower	Upper
{'(Intercept)' }	0.0070033	0.021029	0.33304	20245	0.73911	-0.034214	0.048221
{'type_incon' }	0.039159	0.028477	1.3751	20245	0.16912	-0.016659	0.094977
{'freq_congr20' }	-0.034544	0.025444	-1.3576	20245	0.1746	-0.084417	0.015329
{'normRT' }	0.045973	0.015448	2.976	20245	0.0029241	0.015693	0.076252
{'type_incon:freq_congr20'}	0.049611	0.036892	1.3448	20245	0.17872	-0.0227	0.12192

Random effects covariance parameters (95% CIs):
Group: Subject (35 Levels)

Name1	Name2	Type	Estimate	Lower	Upper
{'(Intercept)' }	{'(Intercept)' }	{'std' }	0.060118	0.034398	0.10507
{'type_incon' }	{'(Intercept)' }	{'corr'}	-0.47669	-0.47843	-0.47495
{'freq_congr20' }	{'(Intercept)' }	{'corr'}	-0.98664	-0.98686	-0.9864
{'normRT' }	{'(Intercept)' }	{'corr'}	0.45293	NaN	NaN
{'type_incon:freq_congr20'}	{'(Intercept)' }	{'corr'}	0.86725	NaN	NaN
{'type_incon' }	{'type_incon' }	{'std' }	0.063685	0.032791	0.12368
{'freq_congr20' }	{'type_incon' }	{'corr'}	0.33119	NaN	NaN
{'normRT' }	{'type_incon' }	{'corr'}	-0.9725	-0.97283	-0.97217
{'type_incon:freq_congr20'}	{'type_incon' }	{'corr'}	-0.81232	-0.81317	-0.81146
{'freq_congr20' }	{'freq_congr20' }	{'std' }	0.088321	0.053873	0.1448
{'normRT' }	{'freq_congr20' }	{'corr'}	-0.30165	NaN	NaN
{'type_incon:freq_congr20'}	{'freq_congr20' }	{'corr'}	-0.79178	NaN	NaN
{'normRT' }	{'normRT' }	{'std' }	0.080471	0.059905	0.1081
{'type_incon:freq_congr20'}	{'normRT' }	{'corr'}	0.73572	0.73499	0.73645

{'type_incon:freq_congr20'} {'type_incon:freq_congr20'} {'std' } 0.089216 0.043214 0.18419

Group: Error
Name Estimate Lower Upper
{'Res Std'} 0.99303 0.98338 1.0028

1c. aMCC

Linear mixed-effects model fit by ML

Model information:
Number of observations 20250
Fixed effects coefficients 5
Random effects coefficients 175
Covariance parameters 16

Formula:
aMCC ~ 1 + normRT + type*freq + (1 + normRT + type*freq | Subject)

Model fit statistics:

AIC	BIC	LogLikelihood	Deviance
57368	57534	-28663	57326

Fixed effects coefficients (95% CIs):

Name	Estimate	SE	tStat	DF	pValue	Lower	Upper
{'(Intercept)' }	-0.063887	0.018441	-3.4643	20245	0.00053263	-0.10003	-0.027741
{'type_incon' }	0.091973	0.027084	3.3959	20245	0.00068547	0.038886	0.14506
{'freq_congr20' }	0.068751	0.020621	3.3341	20245	0.00085727	0.028333	0.10917
{'normRT' }	0.062417	0.0092072	6.7791	20245	1.2425e-11	0.04437	0.080464
{'type_incon:freq_congr20'}	-0.078996	0.034608	-2.2826	20245	0.022462	-0.14683	-0.011163

Random effects covariance parameters (95% CIs):
Group: Subject (35 Levels)

Name1	Name2	Type	Estimate	Lower	Upper
{'(Intercept)' }	{'(Intercept)' }	{'std' }	0.0018855	NaN	NaN
{'type_incon' }	{'(Intercept)' }	{'corr'}	-1	NaN	NaN
{'freq_congr20' }	{'(Intercept)' }	{'corr'}	1	NaN	NaN
{'normRT' }	{'(Intercept)' }	{'corr'}	1	NaN	NaN
{'type_incon:freq_congr20'}	{'(Intercept)' }	{'corr'}	1	NaN	NaN
{'type_incon' }	{'type_incon' }	{'std' }	0.035106	NaN	NaN
{'freq_congr20' }	{'type_incon' }	{'corr'}	-1	NaN	NaN
{'normRT' }	{'type_incon' }	{'corr'}	-1	NaN	NaN
{'type_incon:freq_congr20'}	{'type_incon' }	{'corr'}	-1	NaN	NaN
{'freq_congr20' }	{'freq_congr20' }	{'std' }	0.0022193	NaN	NaN
{'normRT' }	{'freq_congr20' }	{'corr'}	1	NaN	NaN
{'type_incon:freq_congr20'}	{'freq_congr20' }	{'corr'}	1	NaN	NaN
{'normRT' }	{'normRT' }	{'std' }	0.033065	0.017463	0.062609
{'type_incon:freq_congr20'}	{'normRT' }	{'corr'}	1	NaN	NaN

{'type_incon:freq_congr20'} {'type_incon:freq_congr20'} {'std' } 0.045454 NaN NaN

Group: Error
Name Estimate Lower Upper
{'Res Std'} 0.99606 0.9864 1.0058

1d. preSMA

Linear mixed-effects model fit by ML

Model information:
Number of observations 20250
Fixed effects coefficients 5
Random effects coefficients 175
Covariance parameters 16

Formula:
preSMA ~ 1 + normRT + type*freq + (1 + normRT + type*freq | Subject)

Model fit statistics:
AIC BIC LogLikelihood Deviance
57236 57402 -28597 57194

Fixed effects coefficients (95% CIs):
Name Estimate SE tStat DF pValue Lower Upper
{'(Intercept)' } -0.045475 0.018451 -2.4646 20245 0.013723 -0.08164 -0.0093097
{'type_incon' } 0.08645 0.026551 3.256 20245 0.0011318 0.034408 0.13849
{'freq_congr20' } 0.018704 0.020754 0.90123 20245 0.36748 -0.021975 0.059384
{'normRT' } 0.075833 0.010066 7.5338 20245 5.1514e-14 0.056104 0.095563
{'type_incon:freq_congr20'} 0.06119 0.034831 1.7568 20245 0.078971 -0.0070812
0.12946

Random effects covariance parameters (95% CIs):
Group: Subject (35 Levels)
Name1 Name2 Type Estimate Lower Upper
{'(Intercept)' } {'(Intercept)' } {'std' } 0.0097469 0.0010182 0.093306
{'type_incon' } {'(Intercept)' } {'corr'} 0.85993 0.85548 0.86426
{'freq_congr20' } {'(Intercept)' } {'corr'} -0.77056 -0.77779 -0.76312
{'normRT' } {'(Intercept)' } {'corr'} -0.99855 -0.99886 -0.99818
{'type_incon:freq_congr20'} {'(Intercept)' } {'corr'} -0.72036 -0.72902 -0.71148
{'type_incon' } {'type_incon' } {'std' } 0.020113 0.0019557 0.20685
{'freq_congr20' } {'type_incon' } {'corr'} -0.98795 -0.98873 -0.98711
{'normRT' } {'type_incon' } {'corr'} -0.88612 -0.8887 -0.88348
{'type_incon:freq_congr20'} {'type_incon' } {'corr'} -0.26545 -0.2676 -0.26329
{'freq_congr20' } {'freq_congr20' } {'std' } 0.01717 0.004074 0.072362
{'normRT' } {'freq_congr20' } {'corr'} 0.8037 0.80053 0.80683
{'type_incon:freq_congr20'} {'freq_congr20' } {'corr'} 0.11301 0.10787 0.11814
{'normRT' } {'normRT' } {'std' } 0.041009 0.02522 0.066684
{'type_incon:freq_congr20'} {'normRT' } {'corr'} 0.68205 0.67564 0.68835

{'type_incon:freq_congr20'}	{'type_incon:freq_congr20'}	{'std' }	0.053555	0.018257	0.15709

Group: Error

Name	Estimate	Lower	Upper
{'Res Std'}	0.99252	0.98289	1.0023

2. Full LME results presented in Fig 4C and Table 3 for

2a. RT

Linear mixed-effects model fit by ML

Model information:

Number of observations	20250
Fixed effects coefficients	4
Random effects coefficients	140
Covariance parameters	11

Formula:

stimRT ~ 1 + type*before + (1 + type*before | Subject)

Model fit statistics:

AIC	BIC	LogLikelihood	Deviance
2.572e+05	2.5731e+05	-1.2858e+05	2.5717e+05

Fixed effects coefficients (95% CIs):

Name	Estimate	SE	tStat	DF	pValue	Lower	Upper
{'(Intercept)' }	635.57	10.15	62.62	20246	0	615.67	655.46
{'type_incon' }	91.469	5.1289	17.834	20246	0	81.416	101.52
{'before_incon' }	4.2544	2.7981	1.5204	20246	0.12842	-1.2302	9.7389
{'type_incon:before_incon'}	-16.262	5.4079	-3.0071	20246	0.002641	-26.862	-5.662

Random effects covariance parameters (95% CIs):

Group: Subject (35 Levels)

Name1	Name2	Type	Estimate	Lower	Upper
{'(Intercept)' }	{'(Intercept)' }	{'std' }	59.494	47.298	74.834
{'type_incon' }	{'(Intercept)' }	{'corr'}	-0.31665	NaN	NaN
{'before_incon' }	{'(Intercept)' }	{'corr'}	-0.73006	NaN	NaN
{'type_incon:before_incon'}	{'(Intercept)' }	{'corr'}	0.36782	0.16076	0.54387
{'type_incon' }	{'type_incon' }	{'std' }	26.434	19.603	35.645
{'before_incon' }	{'type_incon' }	{'corr'}	0.36876	NaN	NaN
{'type_incon:before_incon'}	{'type_incon' }	{'corr'}	-0.84158	NaN	NaN
{'before_incon' }	{'before_incon' }	{'std' }	7.7177	3.4777	17.127
{'type_incon:before_incon'}	{'before_incon' }	{'corr'}	-0.733	-0.8206	-0.61174
{'type_incon:before_incon'}	{'type_incon:before_incon'}	{'std' }	11.827	4.4053	31.754

Group: Error

Name	Estimate	Lower	Upper

{'Res Std'}	137.76	136.42	139.11

2b. MFT

Linear mixed-effects model fit by ML

Model information:

Number of observations	20250
Fixed effects coefficients	5
Random effects coefficients	175
Covariance parameters	16

Formula:

theta ~ 1 + normRT + type*before + (1 + normRT + type*before | Subject)

Model fit statistics:

AIC	BIC	LogLikelihood	Deviance
57306	57472	-28632	57264

Fixed effects coefficients (95% CIs):

Name	Estimate	SE	tStat	DF	pValue	Lower	Upper
{'(Intercept)' }	-0.029449	0.010977	-2.6828	20245	0.0073061	-0.050964	-0.0079335
{'type_incon' }	0.084693	0.021864	3.8736	20245	0.00010757	0.041838	0.12755
{'before_incon' }	0.029479	0.020303	1.4519	20245	0.14653	-0.010317	0.069274
{'normRT' }	0.045911	0.015653	2.933	20245	0.0033612	0.015229	0.076593
{'type_incon:before_incon'}	-0.038264	0.043529	-0.87905	20245	0.37938	-0.12359	0.047056

Random effects covariance parameters (95% CIs):

Group: Subject (35 Levels)

Name1	Name2	Type	Estimate	Lower	Upper
{'(Intercept)' }	{'(Intercept)' }	{'std' }	0.02753	0.012148	0.06239
{'type_incon' }	{'(Intercept)' }	{'corr'}	-0.83899	-0.84031	-0.83766
{'before_incon' }	{'(Intercept)' }	{'corr'}	-0.96609	-0.96696	-0.96519
{'normRT' }	{'(Intercept)' }	{'corr'}	0.15522	NaN	NaN
{'type_incon:before_incon'}	{'(Intercept)' }	{'corr'}	0.40516	NaN	NaN
{'type_incon' }	{'type_incon' }	{'std' }	0.066765	0.034355	0.12975
{'before_incon' }	{'type_incon' }	{'corr'}	0.7296	NaN	NaN
{'normRT' }	{'type_incon' }	{'corr'}	-0.39378	NaN	NaN
{'type_incon:before_incon'}	{'type_incon' }	{'corr'}	-0.79501	-0.79792	-0.79206
{'before_incon' }	{'before_incon' }	{'std' }	0.057046	0.024408	0.13333
{'normRT' }	{'before_incon' }	{'corr'}	0.10382	0.097135	0.11049
{'type_incon:before_incon'}	{'before_incon' }	{'corr'}	-0.34497	NaN	NaN
{'normRT' }	{'normRT' }	{'std' }	0.081888	0.061382	0.10924
{'type_incon:before_incon'}	{'normRT' }	{'corr'}	0.14998	0.14066	0.15928
{'type_incon:before_incon'}	{'type_incon:before_incon'}	{'std' }	0.14207	0.074356	0.27147

Group: Error

Name	Estimate	Lower	Upper
{'Res Std'}	0.99302	0.98337	1.0028

2c. aMCC

Linear mixed-effects model fit by ML

Model information:

Number of observations	20250
Fixed effects coefficients	5
Random effects coefficients	175
Covariance parameters	16

Formula:

aMCC ~ 1 + normRT + type*before + (1 + normRT + type*before | Subject)

Model fit statistics:

AIC	BIC	LogLikelihood	Deviance
57373	57539	-28665	57331

Fixed effects coefficients (95% CIs):

Name	Estimate	SE	tStat	DF	pValue	Lower	Upper
{'(Intercept)' }	-0.0070781	0.010189	-0.69467	20245	0.48727	-0.02705	0.012893
{'type_incon' }	0.046705	0.019702	2.3705	20245	0.017772	0.0080868	0.085323
{'before_incon' }	-0.006867	0.018276	-0.37574	20245	0.70711	-0.042689	0.028955
{'normRT' }	0.060958	0.0090969	6.701	20245	2.1244e-11	0.043128	0.078789
{'type_incon:before_incon'}	-0.060135	0.04042	-1.4878	20245	0.13683	-0.13936	0.019091

Random effects covariance parameters (95% CIs):

Group: Subject (35 Levels)

Name1	Name2	Type	Estimate	Lower	Upper
{'(Intercept)' }	{'(Intercept)' }	{'std' }	0.012735	0.0017107	0.094809
{'type_incon' }	{'(Intercept)' }	{'corr'}	-0.92627	NaN	NaN
{'before_incon' }	{'(Intercept)' }	{'corr'}	-0.91132	NaN	NaN
{'normRT' }	{'(Intercept)' }	{'corr'}	0.22952	NaN	NaN
{'type_incon:before_incon'}	{'(Intercept)' }	{'corr'}	0.32633	NaN	NaN
{'type_incon' }	{'type_incon' }	{'std' }	0.035923	0.0072908	0.17699
{'before_incon' }	{'type_incon' }	{'corr'}	0.99928	0.99908	0.99944
{'normRT' }	{'type_incon' }	{'corr'}	0.1542	NaN	NaN
{'type_incon:before_incon'}	{'type_incon' }	{'corr'}	-0.6585	-0.66029	-0.65671
{'before_incon' }	{'before_incon' }	{'std' }	0.022047	0.0021151	0.2298
{'normRT' }	{'before_incon' }	{'corr'}	0.19153	NaN	NaN
{'type_incon:before_incon'}	{'before_incon' }	{'corr'}	-0.68655	NaN	NaN
{'normRT' }	{'normRT' }	{'std' }	0.032069	0.017158	0.059937
{'type_incon:before_incon'}	{'normRT' }	{'corr'}	-0.84512	NaN	NaN
{'type_incon:before_incon'}	{'type_incon:before_incon'}	{'std' }	0.10441	0.043712	0.2494

Group: Error

Name	Estimate	Lower	Upper
{'Res Std'}	0.99605	0.98638	1.0058

2d. preSMA

Linear mixed-effects model fit by ML

Model information:

Number of observations	20250
Fixed effects coefficients	5
Random effects coefficients	175
Covariance parameters	16

Formula:

preSMA ~ 1 + normRT + type*before + (1 + normRT + type*before | Subject)

Model fit statistics:

AIC	BIC	LogLikelihood	Deviance
57236	57402	-28597	57194

Fixed effects coefficients (95% CIs):

Name	Estimate	SE	tStat	DF	pValue	Lower	Upper
{'(Intercept)' }	-0.030148	0.010964	-2.7497	20245	0.00597	-0.051638	-0.0086575
{'type_incon' }	0.13144	0.020909	6.2861	20245	3.3215e-10	0.090455	0.17242
{'before_incon' }	-0.0015492	0.021628	-0.071628	20245	0.9429	-0.043942	0.040843
{'normRT' }	0.076324	0.009976	7.6508	20245	2.0872e-14	0.056771	0.095878
{'type_incon:before_incon'}	-0.089294	0.041566	-2.1482	20245	0.031707	-0.17077	-0.0078207

Random effects covariance parameters (95% CIs):

Group: Subject (35 Levels)

Name1	Name2	Type	Estimate	Lower	Upper
{'(Intercept)' }	{'(Intercept)' }	{'std' }	0.027499	0.011683	0.064723
{'type_incon' }	{'(Intercept)' }	{'corr'}	-0.87057	-0.87185	-0.86928
{'before_incon' }	{'(Intercept)' }	{'corr'}	-0.97808	NaN	NaN
{'normRT' }	{'(Intercept)' }	{'corr'}	-0.060635	-0.17978	0.060267
{'type_incon:before_incon'}	{'(Intercept)' }	{'corr'}	0.76832	NaN	NaN
{'type_incon' }	{'type_incon' }	{'std' }	0.055473	0.020814	0.14785
{'before_incon' }	{'type_incon' }	{'corr'}	0.76963	0.76726	0.77198
{'normRT' }	{'type_incon' }	{'corr'}	0.039482	-0.024368	0.10301
{'type_incon:before_incon'}	{'type_incon' }	{'corr'}	-0.93738	NaN	NaN
{'before_incon' }	{'before_incon' }	{'std' }	0.072161	0.036111	0.1442
{'normRT' }	{'before_incon' }	{'corr'}	0.15089	NaN	NaN
{'type_incon:before_incon'}	{'before_incon' }	{'corr'}	-0.70259	-0.71434	-0.69044
{'normRT' }	{'normRT' }	{'std' }	0.040302	0.024703	0.065751
{'type_incon:before_incon'}	{'normRT' }	{'corr'}	-0.26164	NaN	NaN
{'type_incon:before_incon'}	{'type_incon:before_incon'}	{'std' }	0.12039	0.048423	0.29933

Group: Error

Name	Estimate	Lower	Upper
{'Res Std'}	0.99218	0.98254	1.0019
